# A network-assisted co-clustering algorithm to discover cancer subtypes based on gene expression

**DOI:** 10.1186/1471-2105-15-37

**Published:** 2014-02-04

**Authors:** Yiyi Liu, Quanquan Gu, Jack P Hou, Jiawei Han, Jian Ma

**Affiliations:** 1 Department of Bioengineering, University of Illinois at Urbana-Champaign, Urbana, Illinois, USA; 2 Department of Computer Science, University of Illinois at Urbana-Champaign, Urbana, Illinois, USA; 3 Medical Scholars Program, University of Illinois at Urbana-Champaign, Urbana, Illinois, USA; 4 Institute for Genomic Biology, University of Illinois at Urbana-Champaign, Urbana, Illinois, USA

**Keywords:** Cancer subtype, Clustering, Gene expression

## Abstract

**Background:**

Cancer subtype information is critically important for understanding tumor heterogeneity. Existing methods to identify cancer subtypes have primarily focused on utilizing generic clustering algorithms (such as hierarchical clustering) to identify subtypes based on gene expression data. The network-level interaction among genes, which is key to understanding the molecular perturbations in cancer, has been rarely considered during the clustering process. The motivation of our work is to develop a method that effectively incorporates molecular interaction networks into the clustering process to improve cancer subtype identification.

**Results:**

We have developed a new clustering algorithm for cancer subtype identification, called “network-assisted co-clustering for the identification of cancer subtypes” (NCIS). NCIS combines gene network information to simultaneously group samples and genes into biologically meaningful clusters. Prior to clustering, we assign weights to genes based on their impact in the network. Then a new weighted co-clustering algorithm based on a semi-nonnegative matrix tri-factorization is applied. We evaluated the effectiveness of NCIS on simulated datasets as well as large-scale Breast Cancer and Glioblastoma Multiforme patient samples from The Cancer Genome Atlas (TCGA) project. NCIS was shown to better separate the patient samples into clinically distinct subtypes and achieve higher accuracy on the simulated datasets to tolerate noise, as compared to consensus hierarchical clustering.

**Conclusions:**

The weighted co-clustering approach in NCIS provides a unique solution to incorporate gene network information into the clustering process. Our tool will be useful to comprehensively identify cancer subtypes that would otherwise be obscured by cancer heterogeneity, using high-throughput and high-dimensional gene expression data.

## Background

For a given type of cancer, there are often subtypes that harbor unique sets of genomic changes and exhibit different patterns of gene expression [1-5]. Subtype information is critically important to tailor more effective treatments for patients, as varying subtypes often respond disparately to the same treatment. In the past decade, many generic clustering-based approaches have been developed to identify cancer subtypes based on gene expression data. Typically, expression levels of *d* genes measured on *n* samples are presented as a *d* × *n* real-valued matrix with the entries representing the corresponding expression level. A clustering method can be applied to partition the columns/rows of this matrix into different clusters such that items in one cluster have similar expression patterns. The partition of columns offers clues to potential cancer subtypes, while the partition of rows can highlight potentially relevant co-expressed genes. The most popular clustering methods used in cancer subtype identification include hierarchical clustering and *k*-means [6,7]. Recently, a number of other clustering methods have also been developed. Consensus clustering [8] is a clustering framework where the same clustering algorithm is applied to different subsets of the data multiple times. A consensus result is then collected to better describe the similarities between samples. This framework has been widely used in cancer subtype analysis [9]. To address the high-dimensional feature space problem (which is almost always the case for gene expression analysis), the method developed in [10] uses sparse clustering techniques to adaptively select a small set of informative features to cluster the samples. There are also several clustering methods that were specifically designed for cancer subtype clustering. In [11,12], survival time information was used to select survival-associated genes and then the samples were clustered using gene expression. In [13,14], an integrated approach was developed to consider multiple types of omics data (e.g. gene expression, mutation, copy number, methylation) to help identify cancer subtypes. However, we are more interested in only utilizing the gene expression data (which is much more accessible than any other types of omics data) and getting the subtype information of a patient (based on molecular features such as gene expression) without using clinical features (such as survival time). More importantly, most previous studies did not incorporate biological information, particularly molecular interactions networks, into the clustering step. Indeed, network is key to understanding the molecular perturbations in cancer [15,16]. If we consider the interconnection between genes during the clustering process, we have more knowledge of gene interactions at a systems level and may improve our ability to identify cancer subtypes. This will in turn allow us to analyze the perturbation of a group of genes and pathways rather than individual ones to better understand tumor heterogeneity.

The motivation of our work is to develop a method that effectively incorporates molecular interaction networks into the clustering process to improve cancer subtype identification. To this end, one previous work employed information of biological networks during the clustering [17]. The method first defined a network distance based on the proximity of two genes in the network and an expression distance of two genes, and then constructed the overall distance metric as a function of network and expression distance metrics for hierarchical clustering. Another recent work incorporated network information to cluster genotypes and phenotypes based on phenotype-gene association matrix [18]. The authors did so by adding penalty and regularization terms into the clustering objective to keep the final results consistent with clusters obtained from prior knowledge on the disease phenotype similarity network. However, these two approaches are not appropriate for our cancer subtype identification. First, as our goal is to cluster cancer patients (not genes as in [17]), we cannot add a network-based distance in the distance metric defined for patients. Second, network-derived clusters [18] are also difficult to define for patients since there is no network structure linking all the patients (like the phenotype similarity network). Finally, simply combining network proximity-defined gene clusters directly with gene expression clusters may be misleading, since neighboring genes can have entirely different expression patterns.

In this work, we introduce a new co-clustering algorithm to effectively integrate network information with expression variation across samples. We call our method “network-assisted co-clustering for the identification of cancer subtypes” (NCIS). The method first learns a weight for each gene as an indicator of its importance to be used in the clustering. The key idea is that genes regulating many other genes and showing highly variable expression patterns will be considered as more informative in the clustering process. Another important contribution of this work is that we embed the gene weights directly into the co-clustering objective function.

Co-clustering simultaneously clusters both samples and features [19,20]. In co-clustering, similarity is a measure of the coherence of features (e.g. genes) and samples in a bi-cluster, rather than a function of feature pairs or sample pairs. Consequently, it considers the local context and is able to automatically select subsets that share similar attributes [21,22]. The method we utilize in NCIS is based on Semi-Nonnegative Matrix Tri-Factorization (SNMTF) [23,24], a member of the matrix factorization-based clustering family. A common underlying assumption of such co-clustering methods is that there exist cluster centroids that characterize the behavior and trend of cluster members, which is mathematically formed as matrix tri-factorization. Matrix factorization has simple formalization when compared to other methods, and was shown to be useful in gene expression analysis [25,26]. To our knowledge, NCIS is the first method to apply SNMTF to achieve weighted co-clustering in cancer subtype identification.

## Methods

### Method overview

**Figure 1:**
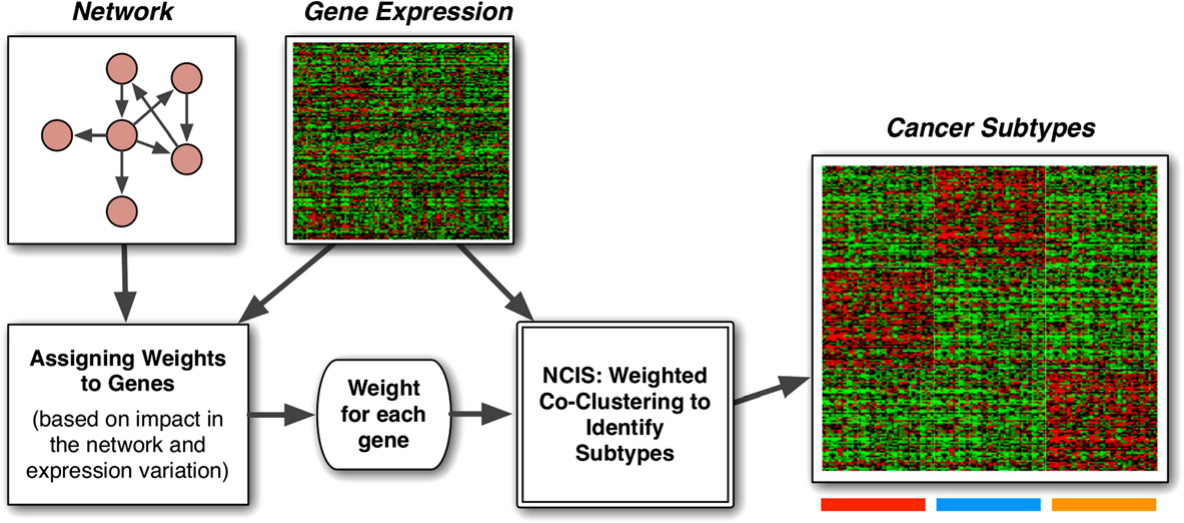
Schematic diagram of our algorithm.

We developed a clustering method that incorporates the gene network (i.e. the interactions between genes) as prior knowledge and simultaneously cluster samples and genes into distinct groups. Adding network structure to the clustering step will help us better select representative genes for clustering. We expect that such a method will generate more biologically informative clusters. The main scheme of our method is shown in Figure 1. Note that we assume the input expression data has already been pre-processed such that the batch effects and technical artifacts are already removed. We implemented the algorithm in MATLAB. Source code and users’ manual can be found at http://bioen-compbio.bioen.illinois.edu/NCIS/.

### Assigning weights to genes

Feature selection is essential for successful pattern recognition from the high-dimensional data. In many previous studies, genes were selected based on their median absolute deviation (MAD) or coefficient of variation (CV) [9,27]. The cutoff was set rather arbitrarily and typically only a small subset of genes was retained for subsequent analysis, which drastically reduces the amount of information used in clustering. Other dimension-reduction methods such as principal component analysis (PCA) [28] are useful, but the biological interpretation is not always straightforward as expression vectors of the samples are projected to a low-dimensional principal component space [29,30]. On the other hand, incorporating additional biologically relevant information as prior knowledge could help resolve ambiguities in the data because it provides, to a certain extent, insight into how the gene expression profiles were generated. Therefore, we utilize the gene network as well as expression information to select genes that both play more important roles in the network and show larger variations among samples. Our method assigns a weight to each gene; genes with higher weights will be prioritized during the weighted co-clustering.

We use a modified PageRank algorithm to assign weights to genes. The original PageRank [31] views the web as a directed graph. Suppose there are *N* nodes (web pages), then *E* is a *N × N* matrix denoting the connections among the nodes. A link from page *i* to page *j* is shown by an edge pointing from node *i* to node *j*, and in the matrix form denoted by *E*_*ij*_ = 1. More links to node *j* raises its confidence level. The algorithm ranks all the nodes based on the iterative formula:

$$r_{j}^{n}=\left(1-\alpha \right)+\alpha \sum_{i=1}^{N}\frac{E_{\mathrm{ij}}r_{i}^{n-1}}{\deg_{i}},1\leq j\leq N,$$

where *r*^*n*^ is the confidence level in the *n*^*th*^ iteration and $\deg_{i}=\sum_{j=1}^{N}E_{\mathrm{ij}}$, referring to the total number of web pages that *i* points to; α is a parameter representing the extent to which the ranking depends on the structure of the graph. In our case, we have a similar network representing the molecular interactions among genes. Our method that assigns weights to genes is similar to a gene ranking method, GeneRank [32], which extended the idea of PageRank to microarray gene differential expression analysis. However, we use a directed graph (rather than undirected graph in GeneRank), because we believe the direction of edges that models gene regulation is important. Additionally, rather than using differential expression, we use an alternative method to consider gene expression variation across samples as described below.

In our graph, a directed edge from node *i* to node *j* means that gene *i* regulates the expression of gene *j*. Genes with larger variations among samples tend to have more distinguishing power. We incorporate such variation into the model to assign weights to genes. Our main idea is that genes having a lot of heavy-weight downstream targets should be assigned large weights – a rationale similar to the confidence vote in the original PageRank, except that outgoing edges increase a gene’s weight while incoming edges increase a web page’s weight. Our weight-training approach is:

$$w_{j}^{n}=\left(1-\alpha \right)\mathrm{NMA}D_{j}+\alpha \sum_{i=1}^{N}\frac{E_{\mathrm{ji}}w_{i}^{n-1}}{\deg_{i}},1\leq j\leq N,$$

where $\deg_{i}=\sum_{j=1}^{N}E_{\mathrm{ji}}$ is the total number of genes that regulate gene *i*; *w*^*n*^ is the weight vector of genes in the *n*^*th*^ iteration and *NMAD* is the normalized median absolute deviation (MAD):

$$\mathrm{NMA}D_{i}=\frac{\mathrm{MA}D_{i}}{\max\left(\mathrm{MAD}\right)},$$

where max(*MAD*) is the maximum of vector *MAD*.

We use MAD as a measurement of the expression variation of a gene among all the samples. The values are normalized by the maximum value in all MAD’s to make the weight-training mechanism stable and comparable with different overall expression levels. In each iteration, every gene *i* is evaluated by its own MAD as well as the weights and connections of the genes that *i* regulates. The final weight of each gene reflects both its impact in the network and its ability to separate the samples.

The convergence of this iterative algorithm is guaranteed for any 0 < *α* < 1 [32,33]. Let *w*^*n+*1^ *= w*^*n*^, we have

$$w_{j}^{n}=\left(1-\alpha \right)\mathrm{NMA}D_{j}+\alpha \sum_{i=1}^{N}\frac{E_{\mathrm{ji}}w_{i}^{n}}{\deg_{i}},1\leq j\leq N.$$

We can write in the matrix form as

$$w^{n}=\left(1-\alpha \right)\mathrm{NMAD}+\mathrm{\alpha E}D^{-1}w^{n},$$

where *D* is a diagonal matrix with entries deg_*i*_, 1 ≤ *i* ≤ *N*.

The final weight for all the genes can be represented as:

$$w=\left(1-\alpha \right)\times {\left(I-\mathrm{\alpha E}D^{-1}\right)}^{-1}\times \mathrm{NMA}D,$$

where *I* is the *N* × *N* identity matrix.

To make weights trained with different parameters more comparable, we normalized *w* such that the maximum of *w* is 1. We chose a relatively large *α* value (*α* = 0.85) to make the weights rely more on the network structure.

### Weighted co-clustering algorithm

After assigning weights to genes, our input data include the gene expression profile of each sample and the weights for all the genes from the previous step. We developed a new weighted co-clustering method to simultaneously separate samples into subtypes and group genes into functionally relevant subclasses. Our method is based on Semi-Nonnegative Matrix Tri-Factorization (SNMTF), where the nonnegative constraint on the data matrix imposed on Orthogonal Nonnegative Matrix Tri-Factorization (ONMTF) is relaxed to make it suitable for general dataset.

### Objective

Suppose our gene expression matrix *X* contains *d* genes and *n* samples, and we would like to group the genes into *m* clusters and group the samples into *c* clusters (subtypes). For convenience, the main notations used in the rest of the paper are presented in Table 1. Our method can be specified as minimizing the following objective,

$$\begin{array}{l} ∥X-\mathrm{GS}F^{T}∥{\;}_{W}^{2}=\sum_{i=1}^{d}∥X_{i\cdot }-{\left(\mathrm{GS}F^{T}\right)}_{i\cdot }{∥}^{2}\times W_{\mathrm{ii}}\\ \;\;\;\;=\mathrm{tr}\left(X^{T}\mathrm{WX}-2X^{T}\mathrm{WGS}F^{T}+FS^{T}G^{T}\mathrm{WGS}F^{T}\right). \end{array}$$

**Table 1:** Main notations used in this paper

**Notation**	**Description**
*n*	Number of samples
*d*	Number of genes
*c*	Number of sample clusters
*m*	Number of gene clusters
*X*	Gene expression matrix of size *d × n*
*X*_*∙i*_	The *i*^*th*^ column of *X*, representing the expression of the *i*^*th*^ sample
*X*_*i∙*_	The *i*^*th*^ row of *X*, representing the expression of the *i*^*th*^ gene
*F*	Sample partition matrix of size *n × c*; *F*_*ij*_ ∈[0,1]: *F*_*ij*_ = 1 if *X*_*∙i*_ belongs to sample cluster *j* and *F*_*ij*_ = 0 otherwise
*G*	Feature partition matrix of size *d × m*; *G*_*ij*_ ∈[0,1]: *G*_*ij*_ = 1 if *X*_*i∙*_ belongs to gene cluster *j* and *G*_*ij*_ = 0 otherwise
*S*	A *m × c* matrix
*W*	A *d × d* diagonal matrix; entries are the weights of the genes

Here, *G* denotes the cluster each gene belongs to and *F* denotes the cluster of every sample. Entries of matrix *S* can be treated as centroids of the blocks generated. The aforementioned weights are presented in the diagonal matrix *W*, and we incorporate an “importance indicator” by multiplying the weights to the row (gene) norms. This is to prioritize genes with large weights in the optimization step. Due to difficulties in minimizing the objective with the binary-value constraint on *F* and *G*, we relax *F* and *G* into continuous nonnegative domain as in previous related work [24]. We only require $\sum_{j=1}^{m}G_{\mathrm{ij}}=1,\sum_{j=1}^{c}F_{\mathrm{ij}}=1$. Thus our objective is to minimize:

$$\begin{array}{l} J=\mathrm{tr}\left(X^{T}\mathrm{WX}-2X^{T}\mathrm{WGS}F^{T}+FS^{T}G^{T}\mathrm{WGS}F^{T}\right),\\ \;s.t.G\geq 0,F\geq 0,\sum_{j=1}^{m}G_{\mathrm{ij}}=1,\sum_{j=1}^{c}F_{\mathrm{ij}}=1. \end{array}$$

### Optimization

We set:

$$\frac{\partial J}{\partial S}=0,$$

Then we have:

$$S={\left(G^{T}\mathrm{WG}\right)}^{-1}G^{T}\mathrm{WXF}{\left(F^{T}F\right)}^{-1}.$$

We can get a clearer understanding of *S* from this expression. If *G* and *F* are defined as in Table 1, i.e., 0/1-valued partition matrix, *F*^*T*^*F* should be a *c × c* diagonal matrix, whose entries represent the number of samples belonging to each sample cluster. *G*^*T*^*WG* should be a *m* × *m* diagonal matrix, with entries equal to the total weights of the features (genes) belonging to each of the *m* feature clusters. Similar to the interpretation of *F*^*T*^*F*, *G*^*T*^*WG* can be considered as the weighted total number of features in each feature cluster (taking feature *i* as *w*_*i*_ features when counting the total number). Therefore, (*G*^*T*^*WG*)^-1^*G*^*T*^*WX* represents the feature cluster centroids on the sample space (*n*-dimension) and *XF(F*^*T*^*F)*^*-*1^ represents the sample cluster centroids on the feature space (*d*-dimension). The difference is that all the samples are assumed to have the same weight of 1, while features are assigned different weights *W*. Entries of *S* can be viewed as feature cluster centroids on the sample-centroids space (*c*-dimension) or as sample cluster centroids on the gene-centroids space (*m*-dimension). Therefore, *S* gives the centroids information of the bi-clusters after partitioning.

Now, assume *S* and *G* are fixed. Let *β* ∈ ℝ^*n* × *c*^ be the Lagrangian multiplier for *F*, then Lagrangian function for *F* is

$$L\left(F\right)=J-\mathrm{tr}\left(\beta F^{T}\right).$$

We set:

$$\frac{\partial L\left(F\right)}{\partial F}=0,$$

Using Karush-Kuhn-Tucker condition [34], we have

$${\left(-A^{+}+A^{-}+FB^{+}-FB^{-}\right)}_{\mathrm{ij}}F_{\mathrm{ij}}=0,$$

where *A* = *X*^*T*^*WGS*, *B* = *S*^*T*^*G*^*T*^*WGS*; *M*^+^ and *M*^-^ are the positive and negative of matrix *M* defined as $M^{+}=\frac{\left|M\right|+M}{2},M^{-}=\frac{\left|M\right|-M}{2}$, respectively. Therefore, we obtain the iterating formula for *F*:

$$F_{\mathrm{ij}}\leftarrow F_{\mathrm{ij}}\sqrt{\frac{{\left(A^{+}+FB^{-}\right)}_{\mathrm{ij}}}{{\left(A^{-}+FB^{+}\right)}_{\mathrm{ij}}}}.$$

Similar derivation leads to the iterative formula of *G*:

$$G_{\mathrm{ij}}\leftarrow G_{\mathrm{ij}}\sqrt{\frac{{\left(C^{+}+\mathrm{WG}D^{-}\right)}_{\mathrm{ij}}}{{\left(C^{-}+\mathrm{WG}D^{+}\right)}_{\mathrm{ij}}}},$$

where *C = WXFS*^*T*^*, D = SF*^*T*^*FS*^*T*^.

The iterations decrease the value of the objective function *J*. Convergence of the algorithm can be shown using a typical approach for the convergence proof of NMF-based methods. For more details, see the proof in the (Additional file 1: Supplementary Materials).

Our algorithm is as follows:

•Initialize F and G.

•*While* not convergent and iterations less than a pre-defined value

Update *S* by

*S* = (*G*^*T*^*WG*)^− 1^*G*^*T*^*WXF*(*F*^*T*^*F*)^− 1^;

Update *F* by

$$F_{\mathrm{ij}}\leftarrow F_{\mathrm{ij}}\sqrt{\frac{{\left(A^{+}+FB^{-}\right)}_{\mathrm{ij}}}{{\left(A^{-}+FB^{+}\right)}_{\mathrm{ij}}}};$$

Update G by

$$G_{\mathrm{ij}}\leftarrow G_{\mathrm{ij}}\sqrt{\frac{{\left(C^{+}+\mathrm{WG}D^{-}\right)}_{\mathrm{ij}}}{{\left(C^{-}+\mathrm{WG}D^{+}\right)}_{\mathrm{ij}}}}.$$

### m and c selection

A question raised in almost all clustering methods is how to determine the cluster numbers. There is no agreed-upon solution. Here we utilize an approach that takes advantage of the stochastic property of our algorithm: although NCIS may not converge to the same solution on each run with different initiations, we could expect the results to be very stable if the clustering is strong enough [8,35]. As in [8,35], we ran NCIS 50 times with randomly selected initiations and defined a sample consensus matrix $\bar{M_{s}}$ and a gene consensus matrix $\bar{M_{g}}$. For each run, a *n* × *n* sample connectivity matrix *M*_*s*_ and a *d* × *d* gene connectivity matrix *M*_*g*_ are obtained:

$$M_{s}\left(i,j\right)=\left\{\begin{array}{c} 1,\mathrm{if}\;\mathrm{Sample}\;i\;\mathrm{and}\;\mathrm{Sample}\;j\;\mathrm{belong}\;\mathrm{to}\;\mathrm{the}\;\mathrm{same}\;\mathrm{cluster}\\ 0,\mathrm{otherwise} \end{array}\right)\;\;;$$

$$M_{g}\left(i,j\right)=\left\{\begin{array}{c} 1,\mathrm{if}\;\mathrm{Gene}\;i\;\mathrm{and}\;\mathrm{Gene}\;j\;\mathrm{belong}\;\mathrm{to}\;\mathrm{the}\;\mathrm{same}\;\mathrm{cluster}\\ 0,\mathrm{otherwise} \end{array}\right)\;\;.$$

Consensus matrices $\bar{M_{s}}$ and $\bar{M_{g}}$ are the averages of *M*_*s*_’s and *M*_*g*_’s over 50 runs respectively. The entries range between 0 and 1, where 0 indicates that the corresponding samples (or genes) belong to different clusters in every run and 1 indicates that they belong to the same clusters in all the cases. Therefore, $1-\bar{M}$ offers a new distance metric between the items ($1-\bar{M_{s}}$ for samples and $1-\bar{M_{g}}$ for genes). Similar to [35], we used $1-\bar{M_{s}}$ and $1-\bar{M_{g}}$ to hierarchically cluster samples and genes, and then we define an average cophenetic correlation coefficient $\frac{\rho \left({\bar{M}}_{s}\right)+\rho \left(\bar{M_{g}}\right)}{2}$ to evaluate the stability. Cophenetic correlation coefficient *ρ*(*C*) is defined as the Pearson correlation between distance matrix 1-*C* and the distance matrix induced by the linkage used in hierarchical clustering for re-ordering *C*. If a clustering is stable, the entries would be close to 0 and 1 (two modes), and in the ideal case (only 0 and 1) *ρ*(*C*) would be exactly 1. We observe how the cophenetic correlation coefficients change as *m* and *c* change and select values where the averaged coefficient starts to decrease.

## Results and discussion

We applied NCIS to two large-scale datasets from TCGA as well as simulated datasets to evaluate the effectiveness of our method. We built the network using a variety of sources, including the network used in [36] as well as our up-to-date curated information from Reactome [37], the NCI-Nature Curated PID [38], and KEGG [39]. The network from [36] consisted of inferred gene-interaction from sources of information such as protein interactions, gene co-expression, protein domain interaction, and text-mined interaction described by [40]. To aggregate all of the networks together, all redundant edges were collapsed to single edges. We combined the edges of each of the databases such that a link between any two nodes *A* and *B* exists in the aggregated network if a link between *A* and *B* exists in any of the databases we used. The resulting aggregated network consisted of 11,648 genes and 211,794 edges. Our method assumes that the network is an aggregation of different biological networks, such as protein-protein interaction network, transcriptional regulatory network, and signaling network etc. In the MATLAB implementation of our program, we also allow users to provide other network information as needed.

### Application to TCGA breast cancer dataset

**Figure 2:**
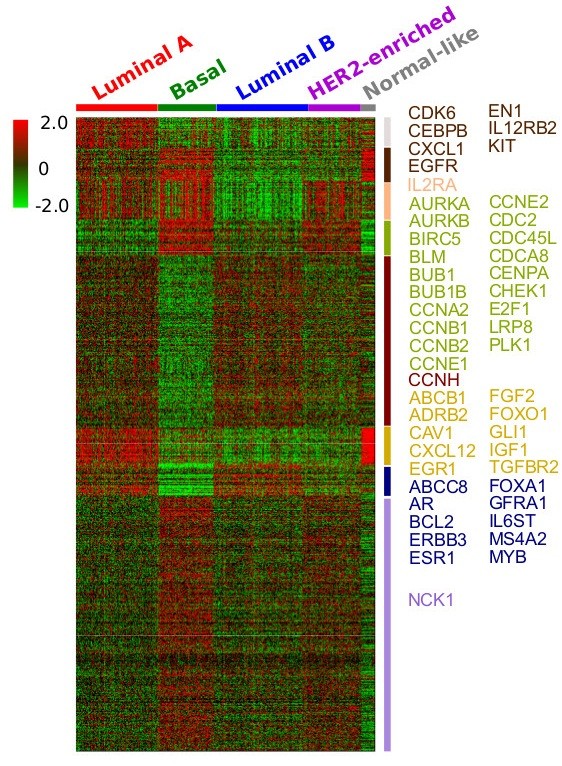
**NCIS result of the TCGA breast cancer expression data.** Genes listed are the first 50 genes shared between the ordered p-value list (based on ANOVA test of each gene’s expression across the five subtypes) and the ordered gene weight list.

The first dataset we used is from a recent large-scale breast cancer study from TCGA [41]. This dataset contains the expression levels of 17,814 genes across 547 samples. We first integrated the gene expression profile with the aggregated network information mentioned above, and trained weights for 8,726 genes included in both of these resources (for genes that are not included in either expression profile or network information, we ignored them). We set α = 0.85 (α is a tuning parameter that represents the extent to which gene weights rely on network structure; see Methods part). The 8,726 weighted genes and 547 samples were the input of NCIS. Figure 2 shows the heatmap with genes and samples rearranged according to the NCIS’s clustering result. Based on the cophenetic correlation coefficient calculated from 50 runs (see Methods part), we chose number of patient clusters *c* = 5 and number of gene clusters m = 8 (Additional file 1: Table S1).

Since we did not know the true class each sample belongs to or the number of subtypes, we used clinical features to evaluate the effectiveness of the clustering algorithm. The underlying idea was that patients in different subgroups were expected to have some different clinical characteristics. We used the following available clinical information to evaluate subtypes identification result: survival time, AJCC staging information (neoplasm disease lymph node stage, neoplasm disease stage and tumor stage) and tumor nuclei percentage. AJCC neoplasm disease lymph node stage represents the stage of cancer based on the lymph nodes present. Neoplasm disease stage represents the extent of a cancer, especially whether the disease has spread from the original site to other parts of the body. Tumor stage is a class assigned to a malignancy which allows for the grouping of similar cancer types based on the extent of disease in the primary tumor, regional lymph nodes, and metastatic sites. Tumor nuclei percentage represents the percentage of tumor nuclei in a malignant neoplasm specimen (from TCGA data dictionary). Table 2 gives the significance level of the difference among all subtypes for each feature. Given p-value threshold 0.05, we conclude that the NCIS (*α* = 0.85)-defined subtypes successfully separated the patients according to these clinical features.

**Table 2:** P-value of the dependence test for different clinical features and breast cancer subtypes

**Method**	**Survival**	**Neoplasm disease lymph node stage**	**Neoplasm disease stage**	**Tumor stage**	**Tumor nuclei percentage**
**NCIS (α = 0.85)**	0.0444	2.03 × 10^-3^	1.68 × 10^-3^	2.33 × 10^-3^	6.17 × 10^-3^
**NCIS (α = 0)**	0.0442	6.22 × 10^-3^	3.84 × 10^-3^	2.67 × 10^-3^	6.24 × 10^-3^
**Consensus (k = 3)**	0.497	0.123	0.266	0.175	5.90 × 10^-3^
**Consensus (k = 5)**	0.359	3.29 × 10^-3^	2.08 × 10^-4^	0.187	8.35 × 10^-3^
**TCGA/BRCA paper**	0.831	0.396	0.337	0.999	0.0780

For survival time, we used logrank test; for AJCC neoplasm disease lymph node stage, AJCC neoplasm disease stage, and AJCC tumor stage, we used Chi-squared test; for tumor nuclei percentage, we used ANOVA. Note that we did not use the normal-like subtype in this comparison.

We also set *α* = 0 (no network information was used) in the co-clustering process to show the impact of network information. Similar statistical tests were performed (Table 2). In general, NCIS (*α* = 0.85) showed better p-values in separating the patients in terms of clinical features than NCIS (*α* = 0).

In the original TCGA paper [41], the authors performed a hierarchical clustering using a subset of genes (most varied across samples) and identified 13 subtypes (test results for clinical features are shown in Table 2 as TCGA/BRCA paper). Since consensus hierarchical clustering generally performs better than the traditional hierarchical clustering, we also applied a consensus average linkage hierarchical clustering [8,42]. To make a fair comparison, we used all 8,726 genes. The program was run over 1,000 iterations and the resampling rate of the sample was set to 0.8. The distance metric was 1 minus Pearson’s correlation coefficient. The algorithm suggested 3 subtypes. However, in Table 2, we listed the tests’ p-values for both 3-subtypes and 5-subtypes conditions to make it easier to compare with the results of NCIS. The results indicated that in general, clusters generated by consensus clustering were not as informative as those from NCIS. We think the most important reason is the lack of effective feature selection in consensus clustering when there are a large number of genes as input.

**Figure 3:**
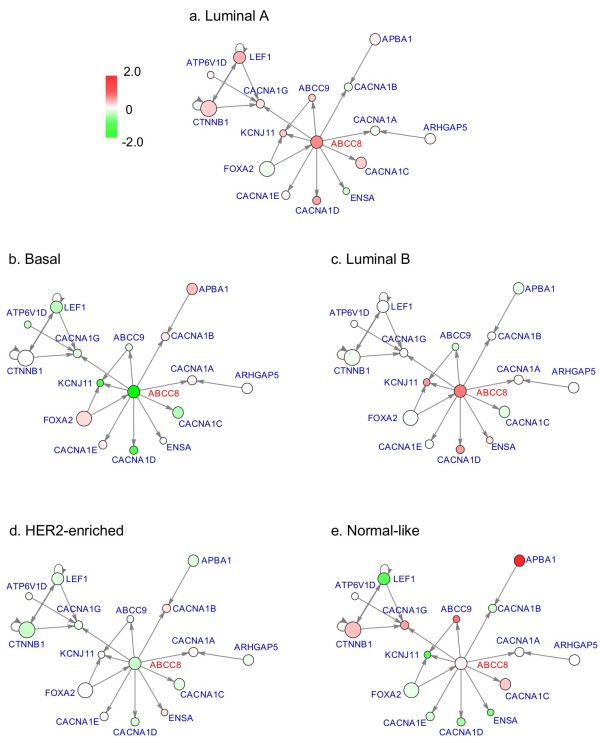
**Expression patterns of ABCC8 subnetwork in breast cancer subtypes.** Genes directly connected to ABCC8 and genes targeting ABCC8's downstream genes are included. Color of circle corresponds to gene expression level; size of circle corresponds to gene weight. **(a)** Subtype Luminal A; **(b)** Subtype Basal; **(c)** Subtype Luminal B; **(d)** Subtype HER2-enriched; **(e)** Subtype Normal-like.

The advantage of NCIS is the incorporation of the network and assigning an “importance indicator” to each gene. Therefore, in addition to generating the subtypes, we also obtained a bi-product -- the gene weights, which describe the genes’ roles in the network and their abilities to distinguish the patient samples. We further performed ANOVA tests for each gene’s expression level across the five subtypes. In the heatmap in Figure 2, we showed the first 50 genes that are shared between the ordered p-value list and the ordered gene weight list (p-values are ordered from smallest to largest and gene weights are ordered from largest to smallest). To illustrate the difference for specific genes in the five subtypes at network level, we extracted the subnetwork of ABCC8 as an example (Figure 3). There are 9 genes targeted by ABCC8 in the network we used. We chose this subnetwork because it has a small size and is easily and clearly presented. Although we did not find literature studying the effect of ABCC8 in breast cancer, MRP has been reported to be highly associated with breast cancer tumor progression and treatment outcomes [43-45]. As shown in Figure 3, ABCC8 is highly expressed in Luminal A and Luminal B subtypes. Several of its downstream genes have very similar expression pattern. Such examples demonstrate the differential expression pattern between subtypes at the network level and the advantage of prioritizing genes with higher impact in the network.

The running time of NCIS (*α* = 0.85, *c* = 5, *m* = 8) on an 8GB memory desktop for this dataset is about 5 minutes.

### Application to TCGA GBM dataset

The second dataset we used was from a recent large-scale TCGA Glioblastoma Multiforme (GBM) subtype identification work [9]. This dataset contains the expression of 11,861 genes on 200 GBM and 2 normal brain samples. In the original paper, the authors first selected 1,903 variably expressed genes according to the MAD and applied consensus hierarchical clustering with agglomerative average linkage [8]. Four subtypes were detected.

We integrated the gene expression data with the network information to train a weight for each of the 7,183 genes included in both sets (network and expression). Tuning parameter α was set to 0.85. After obtaining the weights, these 7,183 weighted-genes and the 202 samples were used in NCIS (result in Figure S1). We set *m* = 7 and *c* = 4 (Additional file 1: Table S2).

We again used clinical characteristics to evaluate the effectiveness of our method. We used survival time, tumor necrosis percentage, and tumor nuclei percentage. Tumor necrosis percentage represents the percentage of cell death in a malignant tumor specimen (from TCGA data dictionary). Additional file 1: Table S3 provides the significance level of the difference among all subtypes for each feature. We also ran consensus average linkage hierarchical clustering [8,42] on the 7,183-gene dataset. The program was run over 1,000 iterations and the resampling rate of the samples was set to 0.8. The distance metric is 1 minus Pearson’s correlation coefficient. We identified 4 subtypes. Overall, NCIS (*α* = 0.85) performed the best. Interestingly, we observed that Subtype Proneural has a much higher survival rate than the other three subtypes (Additional file 1: Figure S2). The underlying mechanism requires more study. In the heatmap in Figure S1, we also showed the first 50 genes that are shared between the ordered p-value list (based on ANOVA test of each gene’s expression across the four subtypes) and the ordered gene weight list. Figure S3 shows the subnetwork of C1QA, which is involved in immune response, to illustrate the difference among subtypes at network level.

The running time of NCIS (*α* = 0.85, *c* = 4, *m* = 7) on an 8GB desktop for this dataset is about 2 minutes.

### Evaluation by simulation

To further assess the performance of NCIS, we simulated a dataset with 300 samples and 3 subgroups. We designed a method to simulate gene expression data based on network interaction structure (see Additional file 1: Supplementary Method). For the 3 subtypes we simulated, the mean expression levels of each gene were estimated from the gene expression profiles of Luminal A, Luminal B, and Basal subtypes in the breast cancer dataset. The final datasets contained 300 samples and 8,726 genes.

To make the simulated datasets more realistic, noisy genes were added. We first trained a weight for each gene based only on the network structure and then chose *l* genes with lowest weights as “uninformative” genes. We randomly permutated the expression levels of these genes across the samples. *l* was set to 1000, 2000, 3000, 4000, and 5000 (we generated 5 datasets for each *l*).

**Figure 4:**
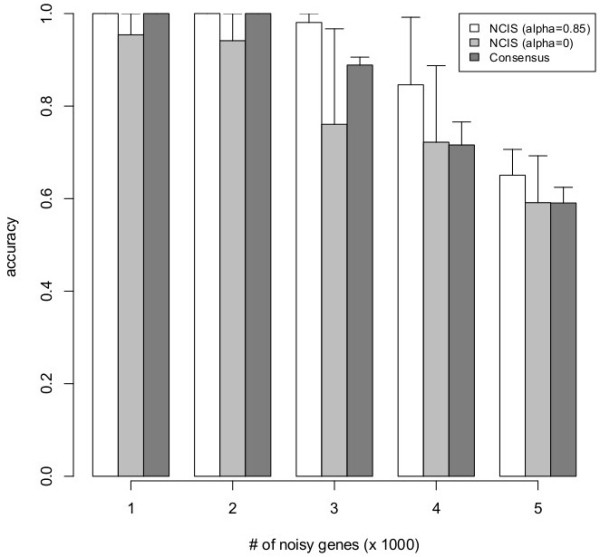
**Accuracies on simulated datasets.** NCIS (*α* = 0.85) vs. NCIS (*α* = 0) vs. consensus clustering on simulated datasets. Height of the solid boxes reflects the average accuracy in each setting (over 5 independent datasets simulated under the setting) and the bar indicates the standard deviation. P-value of paired one-sided t-test (25 data points for each group) for H_0_: Accuracy (NCIS (*α* = 0.85)) ≤ Accuracy (NCIS (*α* = 0)) is 0.0057. P-value of paired one-sided t-test (25 data points for each group) for H_0_: Accuracy (NCIS (*α* = 0.85)) ≤ Accuracy (Consensus clustering) is 0.0019.

We set *m* = 8 and *c* = 3 in NCIS. The results for multiple trials of the simulation studies were shown in the Figure 4. The running time of NCIS (*α* = 0.85, *c* = 5, m = 8) on an 8GB desktop is about 3 minutes for each simulated dataset. We found that when the number of “noisy” genes is small (1000 and 2000), both NCIS (α = 0.85) and consensus-clustering have 100% accuracy. When the number of noisy genes is increased to 3000, NCIS (α = 0.85) starts to perform better than consensus clustering. As expected, once the number of noisy genes becomes excessive (5000 out of 9000), neither method can achieve high accuracy. Overall, our simulation result indicated that NCIS is a more robust method than consensus clustering to tolerate noise.

We also observed that NCIS (α = 0.85) outperformed NCIS (*α* = 0) significantly in our simulated dataset (Figure 4). However, in the two real datasets, the advantage is marginal. We think the main reason could be that in our simulated datasets, the expression levels are strongly related to the network structure we collected (i.e. the interaction network well reflects how the gene expression is generated), while in real datasets there are more uncertainties and the network information we used is incomplete.

## Conclusions

Cancer subtype information is of critical importance in designing better treatment strategies. We developed a novel clustering method, called NCIS, to identify cancer subtypes from high-throughput gene expression data. NCIS incorporates the network information within the clustering step to detect more informative sample subtypes. NCIS assigns a weight to each gene based on its connection in network and its distinguishing ability in expression level across all samples. Our approach avoids excluding a large number of genes, which results in much information loss for subsequent analysis in previous methods. In addition, we utilize a weighted co-clustering method to capture the duality of gene expression data, i.e. similarity is treated as a level of coherence of the samples and genes in the bi-cluster.

The future directions of this problem should ideally address three key challenges. First, the network we used is assumed to be a generic molecular interaction network; it is not specific for the particular type of cancer or the tissue-type. Second, the network does not contain all the genes. Third, many edges in our current network do not have a high confidence level and the directions of many edges are unclear. These three problems can be addressed as we gain more complete understanding of the network.

Further research is needed to design better approaches to choose the optimal parameters in NCIS, including α, *c*, and *m*. Since there is often no gold standard available for the clustering problem of a specific type of cancer, it is difficult to find the optimal parameters of *α*, *c*, and *m*. In our study, we use *α* = 0.85 to keep the balance between network information and gene expression information. We did test the results using different values of *α*, such as 0.8 and 0.7, and the results were comparable with minor differences in the clustering result. We believe the problem of choosing the optimal *α* may require further studies when more data is collected through large-scale projects with detailed clinical features in the future. Such knowledge can be utilized to help select *α*. Additionally, how to determine the number of clusters (*c* and *m*) remains a difficult problem in clustering algorithms. In our work, we utilized cophenetic correlation coefficients used in [35]. We compared the results using different *m* and *c* combinations where the cophenetic coefficients are slightly lower than the optimal combination (Additional file 1: Table S1 and S2). For both BRCA and GBM data, we observed that the subtypes identified were very similar (i.e., they had high correlation with the results from optimal combination) based on the p-values of a Chi-squared test between subtypes identified in the optimal combination. Therefore, the small variation in the choices of *m* and *c* results in very similar clustering here. However, further research is still needed to develop better approaches to automatically select the most appropriate *m* and *c*.

We believe our new NCIS algorithm will be useful to comprehensively identify cancer subtypes, which would otherwise be obscured by cancer heterogeneity, using high-throughput and high-dimensional gene expression data. Results from NCIS are expected to enhance our ability to discover important subtype patterns and key genes involved in each subtype, which will in turn help us better understand important network perturbations in a subtype-specific manner.

## Competing interests

The authors declare that they have no competing interests.

## Authors’ contributions

YL, QG, JH, and JM conceived the research. YL developed the algorithm and performed the data analysis with contributions from QG and JHP. YL drafted the manuscript. QG, JPH, JH, and JM revised the manuscript. All authors read and approved the final manuscript.

## Supplementary Material

Additional file 1 **Supplementary materials.** Available at *BMC Bioinformatics* online. Click here for file
