# Supplementary material for: A network-assisted co-clustering algorithm to discover cancer subtypes based on gene expression
Source: BMC Bioinformatics. 2014 Feb 4;15:37. doi: 10.1186/1471-2105-15-37 (PMC3916445; doi:10.1186/1471-2105-15-37)
Supplement: Additional file 1 — Supplementary materials. Available at BMC Bioinformatics online. [file 1471-2105-15-37-S1.pdf]

SUPPLEMENTARY MATERIALS

Yiyi Liu, Quanquan Gu, Jack P. Hou, Jiawei Han, and Jian Ma

Proof of Convergence

We use the auxiliary function approach to prove the convergence of our algorithm.

$Z(h, h')$ is called an auxiliary function for $F(h)$ if $Z(h, h') \geq F(h)$ and $Z(h, h) = F(h)$. Let $h^{(t+1)} = \arg\min_h Z(h, h^{(t)})$, then $F(h^{(t+1)}) \leq Z(h^{(t+1)}, h^{(t)}) \leq Z(h^{(t)}, h^{(t)}) \leq F(h^{(t)})$.

Lemma 1. *For any matrices $U \in \mathbb{R}_+^{k\times k}$, M, $M' \in \mathbb{R}_+^{n\times k}$, if U is symmetric, the following inequality holds:*

$$\sum_{i=1}^{n}\sum_{j=1}^{k}\frac{(M'U)_{ij}M_{ij}^2}{M'_{ij}} \geq tr(M^T MU). \tag{1}$$

Proof. Let $M_{ij} = v_{ij}M'_{ij}$.

$$LHS = \sum_{i=1}^{n}\sum_{j,l=1}^{k} M'_{il}U_{lj}M'_{ij}v_{ij}^2 = \sum_{i=1}^{n}\sum_{j,l=1}^{k} M'_{il}U_{lj}M'_{ij}\frac{v_{ij}^2 + v_{il}^2}{2}.$$

$$RHS = \sum_{i=1}^{n}\sum_{j,l=1}^{k} v_{il}M'_{il}v_{ij}M'_{ij}U_{jl} = \sum_{i=1}^{n}\sum_{j,l=1}^{k} M'_{il}U_{lj}M'_{ij}v_{ij}v_{il}.$$

Therefore,

$$LHS - RHS = \sum_{i=1}^{n}\sum_{j,l=1}^{k} M'_{il}U_{lj}M'_{ij}\frac{(v_{ij} - v_{il})^2}{2} \geq 0.$$

□

For any real-valued matrix A, define $A^+ = \frac{|A|+A}{2}, A^- = \frac{|A|-A}{2}$.

Theorem 1. *Let $J(M) = tr(-2PM^T + MQM^T)$, where $P \in \mathbb{R}^{n\times k}$ and $Q \in \mathbb{R}^{k\times k}$ are fixed matrices, and Q is symmetric, $M \in \mathbb{R}^{n\times n}$. Then*

$$\begin{aligned} Z(M, M') = &-2\sum_{i,j} P_{ij}^+ M'_{ij}(1 + \log\frac{M_{ij}}{M'_{ij}}) + \sum_{i,j} P_{ij}^- \frac{M_{ij}^2 + {M'_{ij}}^2}{2M'_{ij}} \\ &+ \sum_{i,j}\frac{(M'Q^+)_{ij}M_{ij}^2}{M'_{ij}} - \sum_{i,j,l} Q_{jl}^- M'_{ij}M'_{il}(1 + \log\frac{M_{ij}M_{il}}{M'_{ij}M'_{il}}) \end{aligned} \tag{2}$$

is an auxiliary function of $J(M)$.

Furthermore, fixing M', Z(M,M') is a convex function of M and it has the global minimum at

$$M_{ij} = M'_{ij}\sqrt{\frac{P^+_{ij} + (M'Q^-)_{ij}}{P^-_{ij} + (M'Q^+)_{ij}}} \tag{3}$$

Proof.

$$\forall x \in \mathbb{R}_+,\ 1 + \log x \leq x$$

$$\Longrightarrow\ P^+_{ij}M'_{ij}(1 + \log\frac{M_{ij}}{M'_{ij}}) \leq P^+_{ij}M_{ij};\ Q^-_{jl}M'_{ij}M'_{il}(1 + \log\frac{M_{ij}M_{il}}{M'_{ij}M'_{il}}) \leq Q^-_{jl}M_{ij}M_{il}$$

$$a^2 + b^2 \geq 2ab \implies P^-_{ij}\frac{M^2_{ij} + {M'_{ij}}^2}{2M_{ij}} \geq P^-_{ij}M_{ij}.$$

$$Lemma1 \implies \frac{(M'Q^+)_{ij}M^2_{ij}}{M'_{ij}} \geq \mathrm{tr}(M^TMQ^+).$$

Therefore,

$$Z(M, M') \geq -2tr(P^+M^T) + 2tr(P^-M^T) + tr(M^TMQ^+) - tr(MQ^-M^T) = J(M).$$

To find the minimum of $Z(M, M')$, we take

$$\frac{\partial Z}{\partial M_{ij}} = -2P^+_{ij}\frac{M'_{ij}}{M_{ij}} + 2P^-_{ij}\frac{M_{ij}}{M'_{ij}} + 2\frac{(M'Q^+)_{ij}M_{ij}}{M'_{ij}} - 2\frac{(M'Q^-)_{ij}M'_{ij}}{M_{ij}},$$

$$\frac{\partial^2 Z}{\partial M_{ij}\partial M_{lm}} = \delta_{il}\delta_{jm}(2P^+_{ij}\frac{M'_{ij}}{M^2_{ij}} + 2\frac{P^-_{ij}}{M'_{ij}} + 2\frac{(M'Q^+)_{ij}}{M'_{ij}} + 2\frac{(M'Q^-)_{ij}M'_{ij}}{M^2_{ij}}) \geq 0.$$

Therefore, $Z(M, M')$ is a convex function of M.

Let $\frac{\partial Z}{\partial M_{ij}} = 0$, we have $M_{ij} = M'_{ij}\sqrt{\frac{P^+_{ij}+(M'Q^-)_{ij}}{P^-_{ij}+(M'Q^+)_{ij}}}$.

Therefore, $\arg\min_M Z(M, M')$ has entries $M'_{ij}\sqrt{\frac{P^+_{ij}+(M'Q^-)_{ij}}{P^-_{ij}+(M'Q^+)_{ij}}}$. □

Let $P = X^TWGS = A$, $Q = S^TGTWGS = B$ and $M = F$, we can see that updating F using

$$F_{ij} = F_{ij}\sqrt{\frac{A^+_{ij} + (FB^-)_{ij}}{A^-_{ij} + (FB^+)_{ij}}} \tag{4}$$

monotonically decreases the value of the objective function J in the method part. Besides, we know that $J \geq 0$, so the updating algorithm converges. Since W is a diagonal matrix with positive entries, we can similarly let $P = XFS^T = W^{-1}C$, $Q = SF^TFS^T = D$ and $M = G$, so updating G using

$$G_{ij} = G_{ij}\sqrt{\frac{C^+_{ij} + (WGD^-)_{ij}}{C^-_{ij} + (WGD^+)_{ij}}} = G_{ij}\sqrt{\frac{(W^{-1}C)^+_{ij} + (GD^-)_{ij}}{(W^{-1}C)^-_{ij} + (GD^+)_{ij}}} \tag{5}$$

monotonically decreases the value of the objective function J, and as $J \geq 0$, the updating algorithm converges.

Supplementary Method

Simulate gene expression based on network

We designed a method to simulate gene expression data based on network interaction structure. We assumed that for sample i in subgroup k, gene expression $x_{\cdot i} \sim N(\mu_k, \Sigma)$, where μ_k is a column vector representing the mean expression levels for subgroup k, and Σ is used to model the network structure. To estimate Σ, we used graph Laplacian of the network. We first obtained an adjacency matrix $\tilde{A}$ from the original network matrix $E : \tilde{A} = \max(E, E')$, E' is the transposal of E. The degree matrix $\tilde{D}$ is defined as a diagonal matrix with entries equal to the sum of the corresponding rows of $\tilde{A}$. The graph Laplacian is thus $L = \tilde{D} - \tilde{A}$. We estimated Σ as:

$$\Sigma = v(I - \tilde{D}^{-\frac{1}{2}} \tilde{A} \tilde{D}^{-\frac{1}{2}})$$

The main idea is that the expression levels of genes connected in the network structure are correlated and that the correlations are proportional to the proximities in the network. We used this technique to simulate datasets where interactions between genes can be considered. To determine a constant v, we compared the diagonal entries of matrix $I - \tilde{D}^{-\frac{1}{2}} \tilde{A} \tilde{D}^{-\frac{1}{2}}$ (expression variance) and the variance of gene expression levels. In our simulation, we set $v = 0.5$.

Supplementary Tables

Table S1: **Cophenetic coefficients for BRCA data**

	$m = 6$	$m = 7$	$m = 8$	$m = 9$
$c = 4$	0.931	0.925	0.930	0.944
$c = 5$	0.930	0.940	0.948	0.942
$c = 6$	0.924	0.929	0.945	0.947
$c = 7$	0.926	0.928	0.942	0.937

c for number of subtypes, m for number of gene clusters.

Table S2: **Cophenetic coefficients for GBM data**

	$m = 6$	$m = 7$	$m = 8$	$m = 9$
$c = 4$	0.911	0.920	0.904	0.916
$c = 5$	0.903	0.904	0.906	0.899
$c = 6$	0.902	0.892	0.902	0.894

c for number of subtypes, m for number of gene clusters.

Table S3: **P-value of the dependence test for different clinical features and GBM subtypes**. For survival time, we used logrank test; for tumor necrosis percentage and tumor nuclei percentage, we used ANOVA. Although GBM paper (Verhaak et al.) also used consensus clustering, we applied consensus clustering to all the 7,183 genes and reported the results in row "Consensus ($k = 4$)"

**Method**	**Survival**	**Tumor necrosis percentage**	**Tumor nuclei percentage**
NCSI ($\alpha = 0.85$)	0.0241	1.14×10^{-4}	3.26×10^{-3}
NCSI ($\alpha = 0$)	0.0140	3.29×10^{-4}	4.28×10^{-3}
Consensus ($k = 4$)	0.101	1.09×10^{-4}	0.0105
GBM paper	0.153	3.25×10^{-5}	8.80×10^{-3}

Supplementary Figures

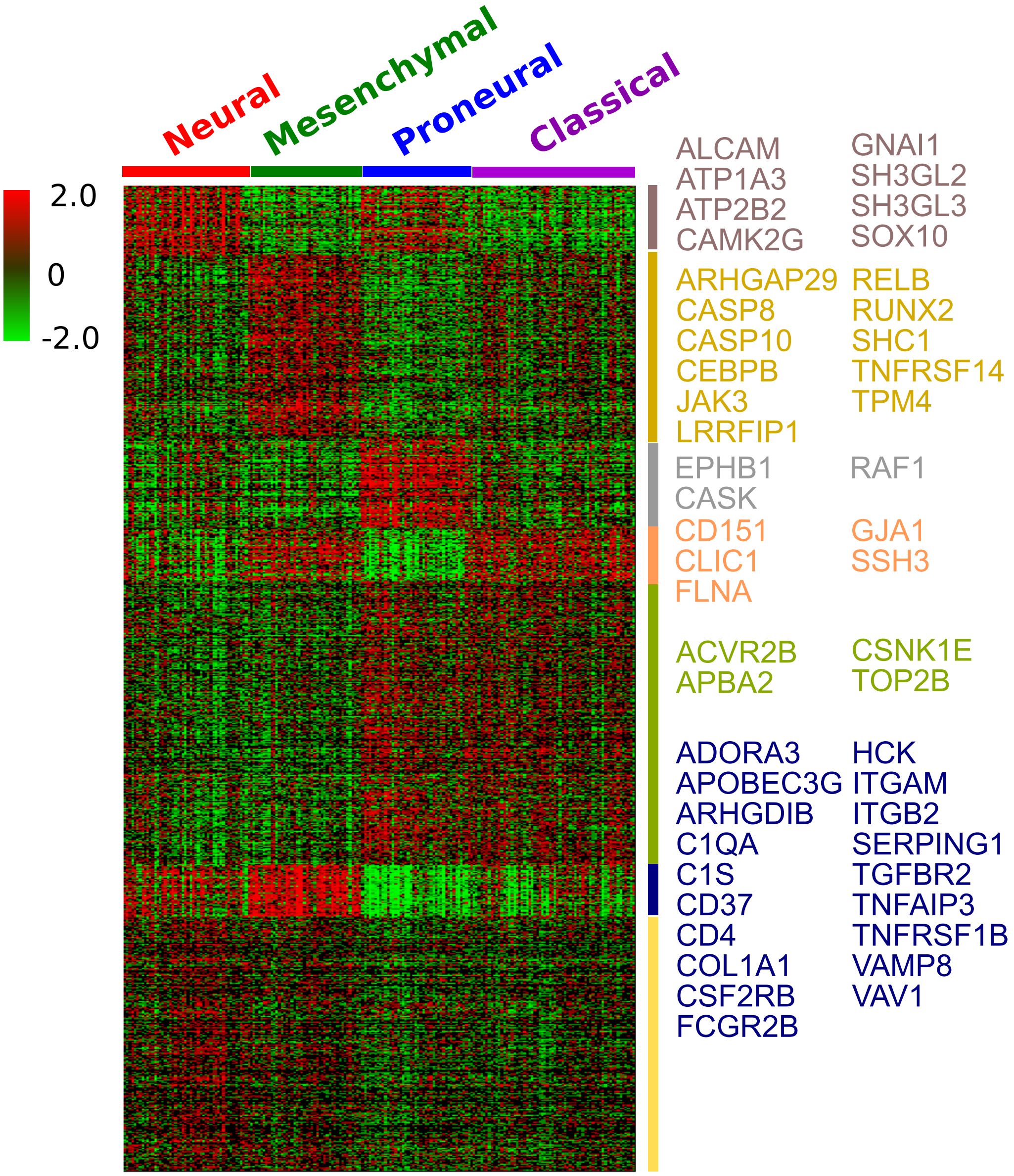

Figure S1: **Heatmap of GBM expression data.** Rearranged according to our NCIS results. Genes listed are the 50 genes that are overlapped in the ordered ANOVA p-value list and the ordered gene weight list.

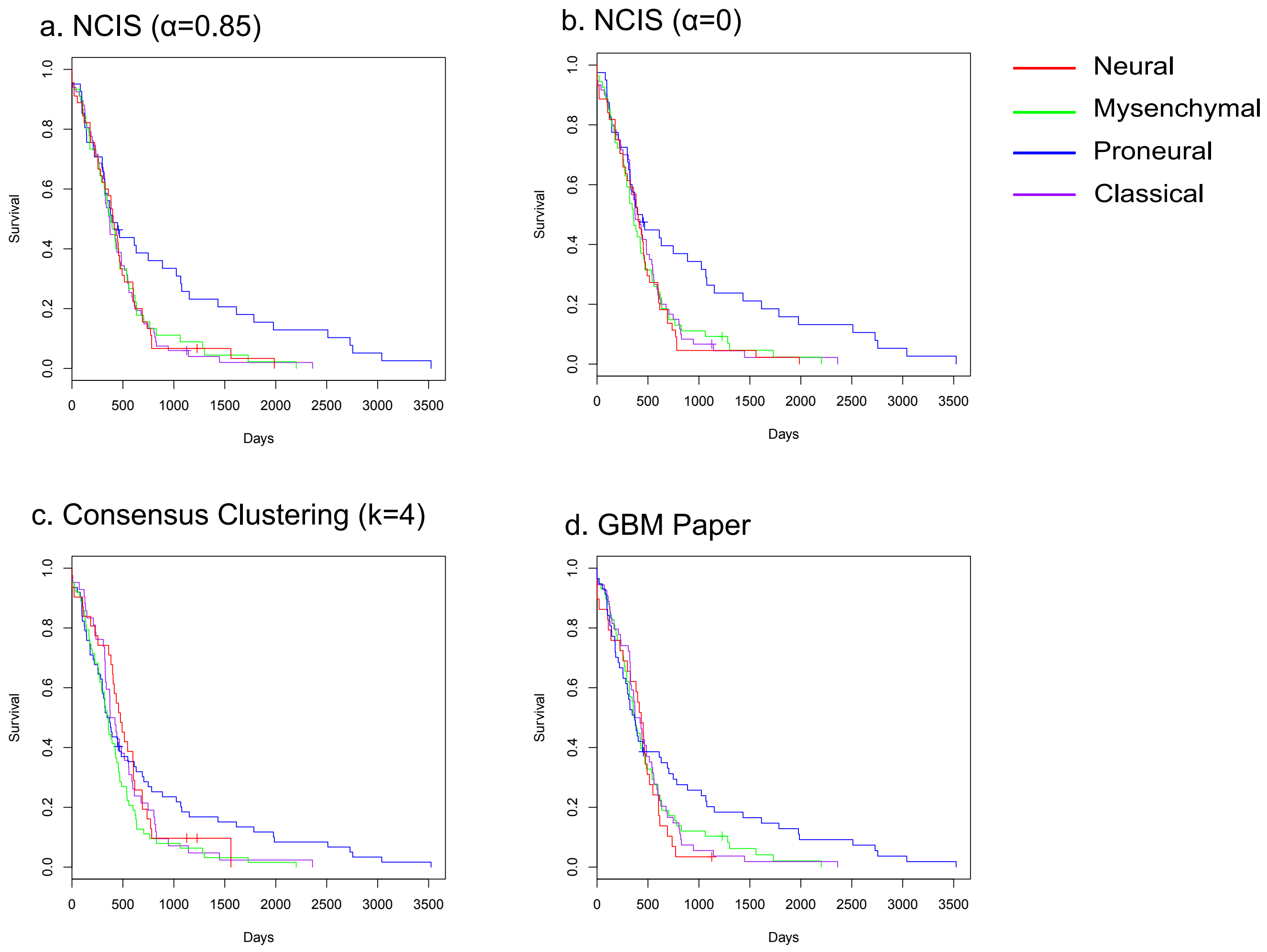

Figure S2: **Kaplan-Meier survival curves of GBM data.** Red for Subgroup Neural, green for Mesenchymal, blue for Proneural and purple for Classical; horizontal axis is the survival time (days) and vertical axis is the survival rate). a. NCIS ($\alpha = 0.85$) defined subtypes; b. NCIS ($\alpha = 0$) defined subtypes; c. Consensus clustering ($k = 4$) defined subtypes; d. GBM paper defined subtypes.

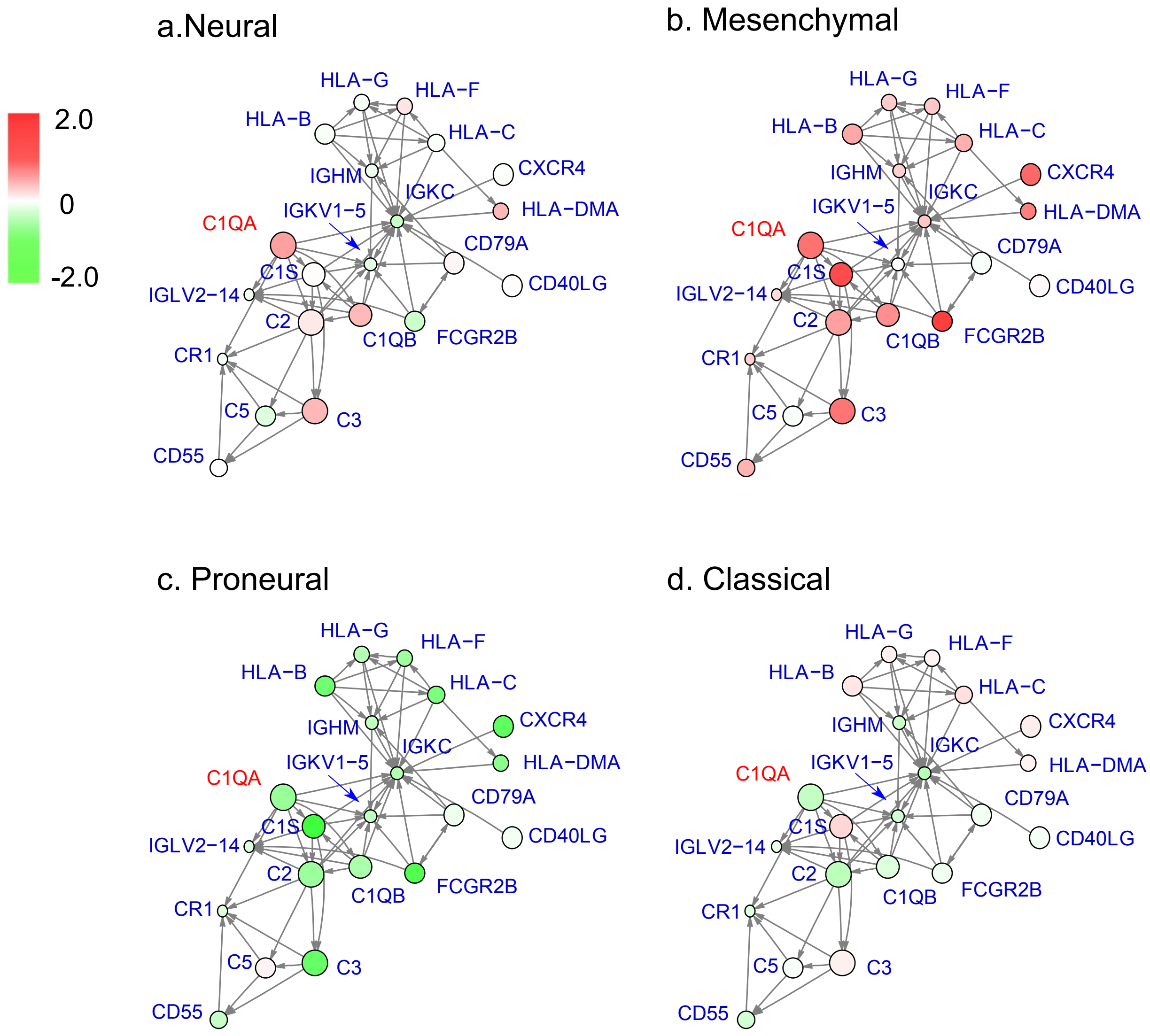

Figure S3: **Expression patterns of C1QA subnetwork in GBM subtypes.** Genes directly connected to C1QA and genes targeting C1QA's downstream genes are included. Color of circle corresponds to gene expression level; size of circle corresponds to gene weight. a. Subtype Neural; b. Subtype Mesenchymal; c. Subtype Proneural; d. Subtype Classical.
